# Building Food Literacy in Adolescence: A Pilot Study of the Teens CAN Curriculum

**DOI:** 10.3390/nu18091434

**Published:** 2026-04-30

**Authors:** Emily Sklar, Tonya Xie, Gretchen L. George, Rebecca Crosby, Marcela D. Radtke, Sheri Zidenberg-Cherr, Rachel E. Scherr

**Affiliations:** 1 Scherr Nutrition Science Consulting LLC, San Francisco, CA 94115, USA; 2 Family, Interiors, Nutrition & Apparel (FINA) Department, San Francisco State University, San Francisco, CA 94132, USA; 3 Department of Epidemiology and Population Health, Stanford School of Medicine, Stanford University, Palo Alto, CA 94305, USA; 4 Department of Nutrition, University of California, Davis, Davis, CA 95616, USA

**Keywords:** food literacy, nutrition education, adolescence, near-peer education, workforce development, curriculum evaluation, real-world application

## Abstract

**Background/Objectives:** There is a limited body of research on evidence-based food literacy education for adolescents. The inquiry-based curriculum, Teens CAN: Comprehensive Food Literacy in Cooking, Agriculture, and Nutrition, was designed to improve food literacy among adolescents ages 14–18 years. This study aimed to assess the Teens CAN curriculum by examining changes in food literacy outcomes among high school–aged adolescents and explore the effectiveness of undergraduate facilitators in implementing the curriculum with fidelity. **Methods:** This quasi-experimental pilot study was conducted among high school students comprising intervention (*n* =14) and comparison groups (*n* = 16). All Teens CAN lessons were delivered by trained undergraduate facilitators, and lesson fidelity was measured by a trained observer. Baseline and follow-up survey measures assessed various components of food literacy, including adolescent nutrition knowledge, diet quality, and intrinsic motivation to prepare healthy food (cooking self-efficacy). Between-group differences were examined using *t*-tests, and ANCOVA regression models assessed associations between changes in baseline to follow-up nutrition knowledge, diet quality, and cooking self-efficacy, adjusting for baseline values. **Results:** The adolescents in the intervention group had a significant increase in nutrition knowledge scores compared to the comparison group (4.6 ± 2.3 vs. 1.1 ± 3.7, respectively; *p* = 0.01). High fidelity (≥80%) was achieved across lessons and lesson components. In ANCOVA regression analyses, participation in the intervention was positively associated with nutrition knowledge (β = 3.3, 95% CI [0.87–5.80]; *p* = 0.01), providing evidence for future investigation. **Conclusions:** The findings from this pilot study suggest that Teens CAN has the potential to positively influence food literacy and related behaviors among adolescents, therefore warranting further investigation in a larger population.

## 1. Introduction

Despite national efforts to improve diet and health-related outcomes among adolescents, recent data indicate that 67% of adolescents within the United States do not meet their dietary recommendations [1,2]. Adolescence, defined by the World Health Organization, is “the phase of life between childhood and adulthood, from ages 10 to 19” [3]. Adolescence marks a unique period of profound physical, emotional, and psychological changes, making it an important stage to establish long-term dietary and health behaviors [3]. One practical approach to promoting healthy eating behaviors in adolescents is through food literacy programming [4,5]. Food literacy is a multidimensional construct encompassing the knowledge, skills, and behaviors required to plan, manage, select, prepare, and eat food to meet needs and shape intake [4]. It is a valuable set of competencies that empowers individuals to make healthy dietary choices [4]. Within this broader framework, nutrition education has emerged as a central and widely used strategy, focusing on building nutrition knowledge as a foundation for behavior change [6]. Nutrition education itself, integrated as part of school or federal-based policies, is an effective approach to positively shape adolescent dietary choices and influence behavioral patterns [7,8,9,10]. However, despite this evidence, the implementation of nutrition education in schools is often hindered by barriers, including limited instructional time, inadequate funding, competing academic priorities, and a lack of trained staff to deliver consistent, high-quality programming [11,12,13,14].

Nutrition, or food literacy education, is also inconsistently integrated into school curricula, especially in communities with limited resources [11]. Structural inequities result in lower resource districts frequently having less access to nutritious food environments and high-quality nutrition programming, further widening existing health disparities and educational achievement gaps among adolescents [15,16,17]. Governmental programs, such as the Supplemental Nutrition Assistance Program Education (SNAP-Ed), have worked to mitigate these inequities by providing free, evidence-based nutrition education to communities with the highest demonstrated need, such as those experiencing high rates of food insecurity and those with low socio-economic status [15,16]. SNAP-Ed partners with state and local organizations to “meet people where they are,” using a unique and effective community-based approach that reflects the needs and assets of the community it serves [18,19]. Despite the demonstrated benefits, recent reductions in funding have resulted in the termination of SNAP-Ed funding and a winddown of programs, exacerbating existing inequities by removing a critical source of nutrition education from students and communities who need it most [20,21,22,23].

Community, however, remains an important influence in shaping adolescent dietary and health-related behaviors [24,25]. A valuable opportunity arises when academic institutions partner with community organizations and local schools to utilize undergraduate students as near-peer facilitators. Engaging undergraduate students as facilitators creates an inexpensive, scalable model that fosters an inclusive environment where lessons are taught through near-peer modeling [26,27,28]. This approach supports adolescents’ learning while expanding access to nutrition education and providing undergraduate students with valuable, often-coveted training and internship experiences that help prepare them for future leadership roles in nutrition and public health. Prior research indicates that leveraging community members, such as undergraduate interns, may provide a practical and sustainable way to expand access to nutrition education, particularly in under-resourced settings [29]. However, nutrition education curricula that leverage community-based approaches and are tailored to adolescents remain limited. Further, while numerous nutrition education programs exist for this population, fewer extend beyond knowledge to incorporate hands-on skill development. In turn, accessible food literacy programming that integrates both knowledge and skill development remains limited [30,31]. Thus, the Teens CAN: Comprehensive Food Literacy in Cooking, Agriculture, and Nutrition curriculum was developed to fill this gap [32].

Teens CAN is an evidence-based food literacy curriculum that was previously developed and refined through content expert review and pilot testing among a subset of high school students in Northern California [32]. The curriculum is grounded in the Social Cognitive Theory and Constructivism to conceptualize behavior change and to capture the interplay between self-efficacy, agency, observational learning, self-regulation, environmental influences, and personal or cognitive factors [33,34]. Backward design guided the curriculum development process, informing the emphasis on cooking, agriculture, and nutrition following an extensive review of the literature [35]. An overview of the Teens CAN lesson components is available in Supplementary Table S1. The final Teens CAN curriculum consists of 12 lessons: four on agriculture, four on nutrition, and four on cooking. Additional details on the curriculum’s development, design, and testing are available in a prior publication [32]. While the curriculum underwent pilot testing to evaluate whether lessons met the learning objectives, a pilot test assessing its impact on food literacy was interrupted due to the COVID-19 pandemic [32]. As a result, the curriculum has not undergone a formative evaluation of its effectiveness and has yet to be implemented in additional research contexts.

Thus, the current study aimed to investigate the impact of Teens CAN and to evaluate its potential for broader implementation across larger populations. To do so, the study focused on understanding how (1) Teens CAN influences food literacy measured by changes in nutrition knowledge, healthy eating behaviors, and motivation to prepare food safely (cooking self-efficacy), and (2) the extent to which Teens CAN supports the development of effective undergraduate nutrition facilitators, as reflected by curriculum delivery fidelity and facilitator self-efficacy.

## 2. Materials and Methods

### 2.1. Recruitment and Participants

This study used a quasi-experimental, pre-post design. Adolescents were recruited using a convenience sampling approach among students attending a public continuation high school in San Francisco, California. This particular school was selected due to its eligibility to participate in CalFresh Healthy Living, University of California (CFHL-UC; SNAP-Ed in California). Recruitment of adolescents was conducted at the classroom level. Classrooms were selected to take part in the study based on teacher interest and ability to integrate into the existing curriculum and thus were not randomly assigned. One Peer Resources class was selected as the intervention group and participated in the full curriculum, while two Spanish classes within the same school served as the comparison group. Adolescents were eligible to participate if they were English-speaking and aged 14–20 years. Parental consent and adolescent assent were obtained for all participants under 18 years old. Adolescents who were 18 years or older provided their own consent. Students within the designated courses were informed that if they did not wish to participate in data collection, they were able to opt out at any time. Data were collected at baseline (pre), prior to administration of the lessons, and again upon completion of the lessons, eight weeks later (post). As the school is a continuation high school serving only juniors and seniors (11th and 12th grade), only adolescents in these grade levels were included. Given that this was a pilot study intended to evaluate the feasibility and efficacy of both the curriculum and the undergraduate facilitator model, a priori power analysis was not performed. As compensation for their time and participation, adolescents received a home hydroponic herb-growing kit and a cooking kit.

#### Data Collection

The evaluation framework of this study was designed to reflect the intervention’s dual aims: improving food literacy and related nutrition outcomes among high school students, as well as assessing the potential of a near-peer, community-engaged model to effectively deliver this curriculum. Thus, measures were selected to assess changes in food literacy-based outcomes among adolescents, as well as fidelity of lesson delivery and self-efficacy. The study underwent full review and was approved by the San Francisco State University Institutional Review Board (IRB protocol number: 2024-108).

### 2.2. High School Students Measures

Pre- and post-questionnaires (baseline and follow-up) were collected via paper surveys distributed to adolescents during class time. The pre- and post-questionnaires were identical, with the exception of the demographics questionnaire, which was only included in the pre-questionnaire. The survey instruments that were included are described below.

#### 2.2.1. Nutrition Knowledge Questionnaire

Nutrition knowledge was assessed using a subset of items drawn from a larger, previously developed, and validated questionnaire designed for high school–aged adolescents; this adolescent questionnaire is an adaptation of the Jones Nutrition Knowledge Questionnaire [36,37]. The original questionnaire contained a total of 49 questions across three content domains: (1) MyPlate recommendations (based on the 2020 Dietary Guidelines for Americans [38]), (2) general nutrition knowledge, and (3) diet–disease relationships. To align with the study population and objectives of the present study, the nutrition knowledge questions were evaluated to ensure relevance and content applicability. Following a priori removal of non-essential questions, the remaining 18 questions were assessed for validity using item-total correlation and for internal consistency reliability using Cronbach-α. Of the 18 questions, two questions did not achieve the minimum item-total correlation of 0.20, indicating poor capability of discrimination between high- and low-scoring individuals, and therefore, those questions were removed from the questionnaire [39]. The Cronbach-α on the remaining 16 questions was 0.76, which is above the minimum acceptable level of α > 0.70 for adequate internal consistency [40]. Following data collection, responses were coded and summed using the devised scoring method, with scores for the Nutrition Knowledge Questionnaire ranging from 0 to 22, where higher scores are indicative of higher knowledge.

#### 2.2.2. Eating and Activity Tool for Students (EATS) Questionnaire

Dietary and physical activity behaviors were assessed using the 21-item Eating and Activity Tool for Students (EATS) Questionnaire [41], which is adapted from the validated School Physical Activity and Nutrition (SPAN) questionnaire [42,43]. This tool was selected for dietary behavior assessment, as it was the tool adopted by the CFHL-UC evaluation for all grade levels across the entire state of California. Adolescents were asked to report their general meal patterns and 24-h intake of key food and beverage groups. Summary scores for total fruit, total vegetable, and total sugar-sweetened beverage intake were calculated based on Likert scale responses. Adolescents were asked how many times they ate specific vegetable groups, beans, fruits, 100% fruit juice, and sugar-sweetened beverages, with one question focused on sodas and the other on sugary fruit-based drinks. With the exception of the fruit question, response options ranged from “No, I didn’t eat/drink ___ yesterday’ up to ‘Yes, I ate/drank ____ 3 or more times yesterday.” Response options for fruit ranged from “No, I didn’t eat fruit yesterday” up to “Yes, I ate fruit 5 or more times yesterday.” A composite healthy dietary behavior score was calculated by adding the number of each of the vegetable groups, beans, fruit groups, and fruit juice and subtracting the number of sugar-sweetened beverages consumed, with scores ranging from −6 to 23. Detailed items and response categories for the EATS questionnaire are described in the original publication [41].

#### 2.2.3. Intrinsic Motivation to Cook Healthy Food Safely Questionnaire (Cooking Self-Efficacy)

Adolescents’ intrinsic motivations, perceived competence, and psychosocial constructs to cook with healthy ingredients such as fruits, vegetables, whole grains, and low-fat milk and dairy products were measured using the previously developed Adolescent Motivation to Cook Healthy Food Safely Questionnaire (cooking self-efficacy questionnaire) [44]. The questionnaire was adapted to include 10 items across 5 subscales: (1) Intrinsic motivation; (2) Perceived competence; (3) Autonomy support; (4) Relatedness; and (5) Autonomy. Possible responses included: “Disagree a lot,” “Disagree,” Neither agree/disagree,” “Agree,” and “Agree a lot.” The 5 sub-scales’ internal reliability was previously assessed using Cronbach’s α coefficients (range, 0.85–0.94). Responses were rated on a 5-point Likert scale from 1 (“Disagree a lot”) to 5 (“Agree a lot”), where higher scores reflected greater cooking self-efficacy.

#### 2.2.4. Demographics

Adolescent demographic information was collected through five questions assessing age, gender identity, race/ethnicity, exposure to tobacco smoke, and self-reported height and weight for BMI calculations.

### 2.3. Delivery of the Intervention

Lessons were delivered using a near-peer teaching model facilitated by five trained undergraduate nutrition interns (4 seniors and 1 junior) enrolled at a large public university in Northern California (San Francisco State University; SFSU) [45,46]. Facilitators were required to be 18 years of age or older and enrolled at SFSU at the time of the study. Facilitators were trained and had structured practice with lesson delivery prior to the start of the intervention. Each lesson was co-taught by two facilitators, one serving as lead and one as an assistant, and overseen by a project lead and the high school teacher. A total of 12 Teens CAN lessons were delivered twice a week from October to December 2024 within 50-min sessions.

### 2.4. Undergraduate Nutrition Facilitators Measure

#### 2.4.1. Fidelity

Fidelity refers to “fidelity of implementation,” which is defined as the degree to which programs are implemented as originally intended by the program developers [47]. In order to ensure the curriculum was appropriately and consistently taught by the undergraduate facilitators, fidelity was measured using a “Program Fidelity Observation Tool” specifically designed for Teens CAN (Supplementary File S1) [48]. Assessments were conducted during the lessons by two SFSU Family and Consumer Science graduate students, who received training in August 2024, and were evaluated to ensure high inter-rater reliability (>90%) of observations [49,50]. Lesson components evaluated include “Opening Questions,” “Procedure,” “Sharing, Processing, Generalizing,” “Follow-Up Questions,” and “Concept or Term Discovery/Introduction.” Other lesson components that were evaluated but not scored include “Questioning strategies,” “Evidence of open-ended questioning,” “Level of youth engagement,” and “Level of adolescent participation compared to leader participation.” Fidelity was scored as follows: “Did not do” = 0 points, “Partially delivered according to curriculum” = 1 point, “Fully delivered according to curriculum” = 2 points. Total sum scores were calculated and expressed as a percentage. The total range of possible scores was 0–10.

#### 2.4.2. Self-Efficacy

Facilitator self-efficacy was assessed using a 10-item retrospective follow-up then baseline questionnaire. Undergraduate facilitators reported on their confidence in teaching others about nutrition, agriculture, and cooking/food safety following their participation in Teens CAN and were then asked to reflect on their confidence prior to program participation. This approach was taken to avoid a response shift bias [48]. Response options were measured on a 5-point Likert scale (“Strongly Disagree,” “Somewhat Disagree,” “Neither Agree nor Disagree,” “Somewhat Agree,” and “Strongly Agree”). Items were summed to generate a total self-efficacy score ranging from 10 to 50, with higher scores indicating greater self-efficacy (Strongly Disagree = 1 to Strongly Agree = 5).

#### 2.4.3. Exit Interview

Upon completion of the intervention, facilitators participated in an informal evaluation-based interview where they described their experiences and provided feedback on the program.

### 2.5. Data Analysis

Descriptive data on participant characteristics assessed at baseline were expressed as absolute (n) and relative frequencies (%) for categorical variables and mean ± standard deviation (SD) for continuous variables. Fisher’s exact tests were used to assess any potential demographic differences between groups. Using baseline and follow-up data, exploratory between-group differences in change scores, the primary outcome of nutrition knowledge scores, and secondary outcomes of dietary intake and cooking self-efficacy were examined using a two-sample *t*-test comparing intervention and comparison groups. Cohen’s d was used to calculate effect sizes for the *t*-tests [51]. To assess changes from baseline to follow-up, while controlling for baseline scores, ANCOVA regressions were conducted. Further, an exploratory, hypothesis-generating regression analysis was used to describe associations between changes in nutrition knowledge and changes in dietary behaviors, adjusting for baseline composite healthy dietary behavior scores. Partial η^2^ as used to measure effect size for the exploratory ANCOVA models, whereas R^2^ was used to assess effect size for the exploratory linear regression model [51]. Data assumptions for parametric tests were evaluated by examining skewness and potential outliers through visual inspection of histograms and boxplots [52]. Normality was assessed using Shapiro–Wilk tests and Q–Q plots [53,54]. Most models met parametric assumptions, with residuals approximately normally distributed. One model showed evidence of non-normality, likely influenced by a small number of higher-value observations. Parametric tests were selected for consistency with past pilot studies, as they offer greater statistical power and allow for estimation of effect size and confidence intervals, which are informative in small pilot studies [55,56]. Given the limited sample size, analyses were exploratory in nature, and results were interpreted cautiously, consistent with recommendations for pilot and underpowered studies [55]. Participants with incomplete data (i.e., missing data or missing timepoints) were excluded from the *t*-test and regression analyses described above. Facilitator fidelity from baseline to follow-up was summarized using percentages and means ± SD. Facilitator self-efficacy scores were reported as absolute (n), relative frequencies (%), and means ± SD. Statistical analysis was performed using R (4.4.1; R Core Team 2024, Posit, PBC, Boston, MA, USA) with significance set at *p* < 0.05.

## 3. Results

A total of 30 adolescents participated in the study (comparison: n = 16; intervention: n = 14). Overall, the mean age of participants was 16.8 ± 0.7 years, with the majority identifying as male, Hispanic, or multiracial/other (Table 1). No statistically significant differences in baseline demographic or health characteristics were observed between comparison and intervention groups. Of the 30 adolescents participating in the pilot intervention, 23 individuals had complete pre- and post-questionnaire data and were included in subsequent exploratory analyses. Due to the nature of the school, attrition in student attendance is not uncommon.

### 3.1. Nutrition Knowledge

Baseline scores were similar between the intervention (n = 9) and comparison (n = 14) groups across outcomes, with no statistically significant differences (Table 2). At follow-up, nutrition knowledge scores were significantly higher in the intervention group than in the comparison group (10.8 ± 3.0 vs. 7.6 ± 3.3, *p* < 0.05) with a mean difference of 3.21 (95% CI: 0.38, 6.03), corresponding to a large effect size (d = 1.00; 95% CI [0.10, 1.88]). Further, individuals in the intervention group demonstrated significantly greater improvements in nutrition knowledge (mean change: 4.6 ± 2.3 vs. 1.1 ± 3.8; *p* = 0.01) over the study period than the comparison group (d = 1.06; 95% CI [0.15, 1.94]).

### 3.2. Cooking Self-Efficacy

Cooking self-efficacy did not significantly differ between groups at baseline or at follow-up. No statistically significant differences in mean change in cooking self-efficacy were observed between groups.

### 3.3. Dietary Outcomes

Among dietary outcomes, the only statistically significant difference observed was for fruit intake at follow-up, which was higher in the comparison group (1.8 ± 0.8 vs. 2.9 ± 1.6; *p* = 0.04), corresponding to a large effect size (d = 0.83; 95% CI [0.05, 1.69]). No statistically significant differences were observed in baseline, follow-up, or in change scores for total fruit and vegetable intake, vegetable intake, or sugar-sweetened beverage intake.

### 3.4. Adjusted Analyses of Study Outcomes

In ANCOVA models adjusting for baseline values of each outcome, the intervention group demonstrated significantly higher nutrition knowledge scores at follow-up compared with the comparison group (β = 3.34, 95% CI [0.87, 5.80], *p* = 0.01, η^2^p = 0.27) (Table 3). No statistically significant differences in cooking self-efficacy or any dietary intake outcomes were observed between groups.

#### Preliminary Exploratory Analyses for Hypothesis Development

Given the small sample size and limited statistical power, exploratory regression analyses were conducted using the generated composite healthy dietary behavior score, adjusting for baseline scores, with the primary aim of generating hypotheses for future research. These preliminary analyses suggested that improvements in nutrition knowledge were positively associated with improvements in dietary behaviors (β = 0.33, 95% CI [0.03, 0.62], *p* = 0.03) with a large effect size (R^2^ = 0.41). However, this finding is not intended to be confirmatory and should be viewed only as a potential relationship to be examined in a larger, adequately powered study.

### 3.5. Facilitator Results

#### Facilitator Fidelity, Self-Efficacy, and Feedback

**Figure 1:**
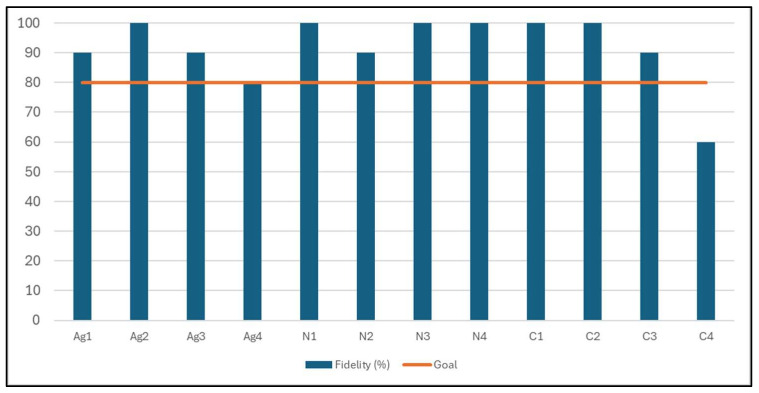
Fidelity scores by lesson across program components.

**Figure 2:**
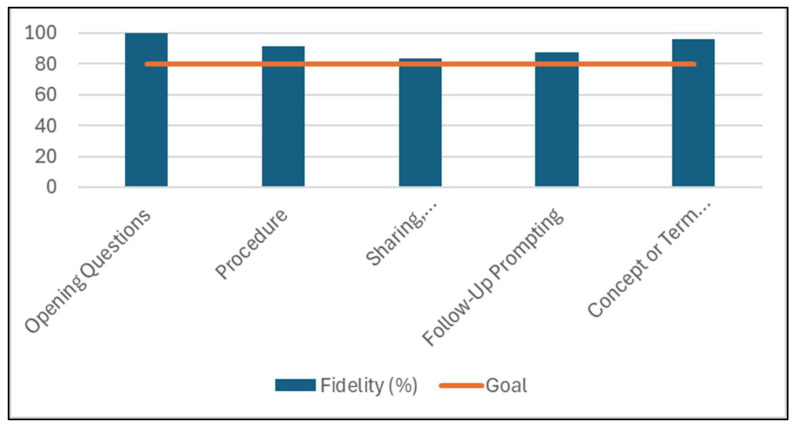
Implementation fidelity by facilitation component.

Overall, mean lesson fidelity was 91.7 ± 14.4%. Lesson fidelity ranged from 60 to 100% (Figure 1). All lessons reached the goal of at least 80% fidelity, other than Cooking lesson 4 (60%). Agriculture lesson 2, Nutrition lessons 1, 3, and 4, and Cooking lessons 1 and 2 were delivered with 100% fidelity. All lesson components (Opening questions; Procedure; Sharing, processing, and generalizing; Follow-up prompting; and Concept or term discovery) reached the goal of 80% fidelity with a mean lesson component fidelity of 91.7% ± 6.6 (Figure 2).

Facilitator responses to the self-efficacy questionnaire are included in Table 4. Facilitators’ self-efficacy scores were 32.6 ± 9.6 prior to the intervention and 44.6 ± 5.1 following the intervention (maximum score = 50). Undergraduate facilitators reported that participation in the program was generally positive and that it provided opportunities to learn more about careers in community nutrition and to develop relevant skills in education and student engagement strategies.

## 4. Discussion

The purpose of this pilot study was to explore the effects of the Teens CAN curriculum on both adolescent and facilitator outcomes, with the goal of understanding the potential efficacy and scalability of the program. Specifically, the study investigated changes in adolescents’ food literacy, comprising nutrition knowledge, dietary behaviors, and intrinsic motivation to prepare healthy food safely (cooking self-efficacy), as well as the extent to which the program enabled effective undergraduate nutrition facilitators. Adolescents participating in the Teens CAN intervention demonstrated greater improvements in nutrition knowledge compared with the comparison group. When examining differences at follow-up and change in nutrition knowledge, participants in the Teens CAN intervention group demonstrated significantly higher nutrition knowledge scores at follow-up and statistically greater changes in knowledge from baseline to follow-up than adolescents in the comparison group. Additionally, when controlling for baseline knowledge, participation in the intervention group was associated with a statistically significant increase in nutrition knowledge when analyzed using regression. Together, these findings suggest that participation in the Teens CAN curriculum may be effective in improving adolescent nutrition knowledge and behaviors. These findings are supported by previous research that demonstrates that food literacy-based interventions can be effective in increasing adolescent nutrition knowledge [31,58]. While the connection between nutrition education interventions and knowledge is well established, it is important to note that knowledge does not always translate to behavior change [24]. For instance, while an individual may have high nutrition knowledge, structural barriers within their environment may be more influential in shaping behavior and dietary habits [24,59]. Thus, in contexts where information is widely and easily accessible, interventions that primarily emphasize the acquisition of knowledge may be limited in their impact on shaping individuals’ behavior [33,60,61]. Instead, focusing on practical real-world applications and decision-making skills may be more effective in supporting meaningful dietary changes [33,60,61]. Using the Social Cognitive Theory as a guiding framework, improvements in knowledge, especially through hands-on learning that develops self-efficacy and skills, may be an influential first step in generating positive dietary behaviors within this population [33,60]. A particular strength of the Teens CAN curriculum is the inclusion of experiential pedagogy that immerses participants in hands-on learning. Additional research in larger adolescent populations is warranted to further examine the role of Teens CAN in shaping nutrition knowledge, skill development, and dietary behavior change and to explore which components of the curriculum are most impactful.

Findings varied when examining behavior change among adolescents. Baseline to follow-up changes in cooking self-efficacy scores and dietary intake measures did not differ significantly by group. However, the absence of detectable changes in dietary intake or self-efficacy may reflect the short intervention duration and limited follow-up period, as well as insufficient power to detect small-to-moderate effects. While no significant changes were seen within this pilot population, prior research evaluating the association between indicators of food literacy, such as nutrition knowledge and self-efficacy, has been shown to be positively associated with dietary outcomes [5,62,63,64]. In the current study, an exploratory analysis was conducted to examine the association between changes in nutrition knowledge and a composite score of dietary behaviors to help generate hypotheses for future research in a larger study population. These analyses were intended to identify patterns that may warrant further investigation and should not be interpreted as definitive findings. Results from the exploratory regression analysis suggested a possible association between greater improvements in nutrition knowledge and more favorable dietary behavior patterns. These exploratory analyses were guided by prior research on adolescent food literacy and related interventions, which have observed similar associations with diet quality and food-related behaviors [58,65,66]. For instance, an intervention that taught adolescents cooking and food preparation skills resulted in increased fruit and vegetable consumption [67]. Similarly, a study evaluating a Culinary Boot Camp food literacy program among undergraduate students reported improvements in cooking skills, self-efficacy, and dietary intake, including increases in micronutrients such as vitamin C, magnesium, and fiber [68]. Further, evidence from recent mixed-methods food literacy interventions targeting adolescents suggests that structured, skills-based programs can produce meaningful short-term improvements, reinforcing the rationale for continued development and evaluation of programs such as Teens CAN [69]. Building on these findings, future research is warranted to explore the sustained impact of the Teens CAN intervention in a larger sample size, using objective measures of diet and behavior where possible, which would improve the statistical power and allow for a more robust estimation of the intervention effects and adolescent outcomes. In order to calculate a future sample size, a change of 1 serving/day of total vegetables was used with an estimated standard deviation of 2.0 servings/day of vegetables. These estimates were selected as recent nationally representative data indicated that adolescent vegetable intake is low and highly variable [70]. Based on this and an α = 0.05 and 80% power, a future study should recruit 75 individuals per group, accounting for a 15% attrition with 63 individuals per group.

Evaluation of the implementation of the lessons indicates that the Teens CAN curriculum was delivered with high fidelity across lessons and facilitators. Almost all lessons met or exceeded the pre-specified fidelity benchmark, with several lessons delivered at full fidelity [71]. One cooking lesson did not meet the set benchmark, which was due to lower engagement related to the instructional focus of the lesson requiring calculations. Additionally, high fidelity was observed across the lesson components. Overall, these findings suggest undergraduate facilitators were able to deliver the curriculum to the adolescents effectively and as intended. As these findings are descriptive and within a small population, they should be interpreted with caution. Feedback from the post-intervention evaluation exit interview indicated that the undergraduates viewed the experience positively and as an opportunity to develop their professional skillset. These findings are supported by prior research, which demonstrates that peer and experiential learning models contribute to greater self-efficacy, skills development, and teaching abilities [72]. The incorporation of undergraduate facilitators was informed by documented benefits of near-peer and experiential learning models for both facilitators and learners, including enhanced self-efficacy, communication skills, decision-making, empathy, and engagement [26,45,73]. Learners may be more likely to engage with educational content when facilitators are perceived as relatable, a key feature of near-peer teaching approaches [28]. From a programmatic perspective, this model also addresses a common challenge in SNAP-Ed and other community health programs that are often limited in resources, particularly with respect to staff time and training capacity. Further, leveraging college students seeking professional development opportunities represents a mutually beneficial strategy to expand program reach while building workforce capacity and parallels the established SNAP-Ed extender model [74]. This approach is especially relevant in the current policy context, as the elimination of SNAP-Ed funding and SNAP-Ed-funded organizations has left a gap in the availability of accurate nutrition education supports, leaving students, schools, and communities without consistent, coordinated nutrition education and related resources [20,21,22,23]. Findings from this study highlight that leveraging near-peer facilitators may help address gaps in nutrition education programming while simultaneously supporting the development of a trained public health nutrition workforce. Future research is needed to examine the impact of participating as a facilitator in the Teens CAN intervention within a larger sample. Further, findings from a larger sample may help identify insights into the competencies required for students to effectively serve as educators and have the potential to inform university course curriculum focused on lesson delivery and educational approaches that better prepare students for leadership roles within similar settings.

### Limitations

As this was a pilot study, the study population was small, resulting in limitations in the analyses and reducing the generalizability and implications that can be made. Further, the self-reported nature of the data collected introduces the potential for response and recall bias [75]. Behavioral outcomes relied on self-reported measures rather than direct observation, which may limit the ability to capture actual behaviors. The pre- and post-questionnaires were self-administered, potentially resulting in bias due to differences in literacy and comprehension. In addition, selection of the intervention class versus the comparison group was completed using convenience sampling based on adolescent and teacher interest rather than random assignment to support the likelihood of intervention completion; however, no significant baseline differences were observed between groups.

## 5. Conclusions

The findings from this pilot study highlight that Teens CAN warrants further exploration within a larger study population to better understand its potential to serve as an impactful, low-cost, community food literacy curriculum for adolescents. Positive associations were observed between nutrition knowledge and improvements in health-promoting dietary behaviors; however, these results are exploratory and should be interpreted accordingly. In addition, the near-peer facilitators delivered the curriculum with high fidelity, demonstrating feasibility within the school setting. Future research examining the implementation of Teens CAN across multiple settings is needed to assess its potential use as a scalable intervention strategy. Taken together, these findings support the potential for the Teens CAN curriculum to support health behavior change among adolescents. Future research with an adequately powered sample is needed to evaluate the effectiveness of Teens CAN across larger, diverse populations and explore long-term, sustained dietary and behavioral outcomes.

## Figures and Tables

**Table 1:** Baseline demographic and health characteristics of study participants by group.

Variables	Characteristics	Total (n = 30)	Comparison (n = 16)	Intervention (n = 14)
Age mean (SD)	Years	16.8 (0.7)	16.6 (0.6)	17.1 (0.6)
Sex n (%)	Male	14 (47)	9 (30)	5 (17)
	Female	11 (37)	5 (17)	6 (20)
	Prefer not to self-identify	5 (17)	2 (7)	3 (10)
Race/Ethnicity n (%)	Non-Hispanic White	5 (17)	3 (10)	2 (7)
	Non-Hispanic Black	5 (17)	3 (10)	6 (20)
	Hispanic	9 (30)	4 (13)	1 (3)
	Asian/Pacific Islander	2 (7)	0 (0)	2 (7)
	Middle Eastern	2 (7)	1 (3)	1 (3)
	Multiracial/Other	7 (23)	5 (17)	2 (7)
BMI mean (SD)	kg/m^2^	23.7 (4.9)	23.7 (4.9)	23.7 (5.2)
Tobacco exposure n (%)	Affirmative Response	7 (23)	2 (7)	5 (17)

**Table 2:** Baseline, follow-up, and differences in nutrition knowledge, cooking self-efficacy, and dietary intake by group.

Time Point	Comparison Mean (SD) (n = 14)	Intervention Mean (SD) (n = 9)	Mean Difference [95% CI]	*p*-Value (Difference Between Groups)
Nutrition Knowledge				
Baseline	6.5 (3.9)	6.2 (3.2)	0.28 [−2.81, 3.37]	0.8
Follow-up	7.6 (3.3)	10.8 (3.0)	3.21 [0.38, 6.03]	0.03
Difference	1.1 (3.8)	4.6 (2.3)	3.48 [0.85, 6.12]	0.01
Cooking Self-Efficacy				
Baseline	34.1 (5.7)	32.4 (5.6)	−1.70 [−6.77, 3.37]	0.5
Follow-up	34.9 (3.7)	34.7 (8.7)	0.19 [−7.02, 6.64]	0.9
Difference	0.7 (4.3)	2.2 (3.9)	1.51 [−2.17, 5.19]	0.4
Total Fruit and Vegetable Intake				
Baseline	5.2 (3.6)	4.0 (1.9)	−1.21 [−3.62, 1.19]	0.3
Follow-up	5.6 (2.6)	5.0 (2.3)	−0.64 [−2.83, 1.55]	0.5
Difference	0.4 (3.0)	1.0 (2.1)	0.57 [−1.66, 2.80]	0.5
Fruit Intake				
Baseline	2.4 (1.9)	1.8 (0.8)	−0.65 [−1.91, 0.61]	0.2
Follow-up	2.9 (1.6)	1.8 (0.8)	−1.15 [−2.23, −0.07]	0.04
Difference	0.5 (1.7)	0 (0.9)	−0.50 [−1.64, 0.64]	0.3
Vegetable Intake				
Baseline	2.8 (2.6)	2.2 (1.6)	−0.56 [−2.39, 1.27]	0.5
Follow-up	2.7 (2.1)	3.2 (2.0)	0.51 [−1.36, 2.38]	0.5
Difference	−0.1 (2.7)	1.0 (2.1)	1.07 [−1.04, 3.18]	0.3
Sugar Sweetened Beverage Intake				
Baseline	2.0 (1.6)	1.6 (1.1)	−0.44 [−1.62, 0.73]	0.4
Follow-up	2.6 (2.2)	1.4 (1.6)	−1.20 [−2.85, 0.45]	0.1
Difference	0.6 (3.0)	−0.1(1.4)	−0.75 [−2.72, 1.21]	0.4

Statistical significance was set at *p* < 0.05. Group differences were assessed using independent *t*-tests.

**Table 3:** Changes in nutrition knowledge, cooking self-efficacy, and dietary intake outcomes by group from baseline to follow-up.

Variable	β [95% CI]	SE	*p* Value
Nutrition Knowledge	3.3 [0.9, 5.8]	1.2	0.01
Cooking Self-Efficacy	1.2 [−2.5, 4.9]	1.8	0.5
Fruit and Vegetables Total Intake	−0.09 [−2.1, 1.9]	0.9	0.9
Fruit Intake	−0.8 [−1.9, 0.2]	0.5	0.1
Vegetable Intake	0.7 [−1.1, 2.5]	0.9	0.4
Sugar-Sweetened Beverage Intake	−1.2 [−3.1, 0.6]	0.9	0.2

Reference group = Comparison Group; Statistical significance *p* < 0.05; ANCOVA with baseline adjustments.

**Table 4:** Facilitator self-efficacy and teaching confidence scores, baseline and follow-up.

Question	Time	Facilitator				
		(1)	(2)	(3)	(4)	(5)
Q1. …I believe I can do a good job teaching students about agriculture.	Baseline	2	3	1	2	5
	Follow-up	5	3	5	4	5
Q2. …I am able to stimulate students enough, so they ask thoughtful questions about agriculture.	Baseline	2	3	1	3	4
	Follow-up	4	3	4	5	5
Q3. …I believe I can do a good job teaching students about nutrition	Baseline	4	4	4	4	5
	Follow-up	5	4	4	5	5
Q4. …I am able to stimulate students enough, so they ask thoughtful questions about nutrition	Baseline	3	4	4	2	5
	Follow-up	5	4	5	4	5
Q5. …I believe I can do a good job teaching students about cooking and food safety.	Baseline	1	4	3	4	5
	Follow-up	5	4	5	5	5
Q6. …I am able to stimulate students enough, so they ask thoughtful questions about cooking and food safety.	Baseline	2	4	2	3	5
	Follow-up	5	4	4	5	5
Q7. …I understand how to lead a group of students through the inquiry-based learning process.	Baseline	1	3	2	1	4
	Follow-up	5	3	5	4	4
Q8. …I believe that using an inquiry-based approach is an effective way to learn and teach.	Baseline	4	4	3	3	5
	Follow-up	4	4	4	4	5
Q9. …I am confident in asking youth open ended questions, such as, “Explain what you know about XX?”	Baseline	5	3	4	2	5
	Follow-up	2	3	5	5	5
Q10. …I believe that participating in a community of practice [57] is an effective way to strengthen educator’s skills.	Baseline	4	4	3	3	5
	Follow-up	5	4	4	4	5

Response options were scored on a 5-point Likert scale (1 = Strongly disagree, 2 = Somewhat disagree, 3 = Neither agree nor disagree, 4 = Somewhat agree, 5 = Strongly agree). At baseline, questionnaire items began with “Prior to participating in the project…,” and at follow-up, questionnaire items began with “After participating in the project…” Grammar was adjusted accordingly.

## Data Availability

The data are available upon request from the corresponding author. The data are not publicly available due to privacy and ethical restrictions.

## Supplementary Materials

The following supporting information can be downloaded at: https://www.mdpi.com/article/10.3390/nu18091434/s1, this will consist of Table S1: Teens CAN Learning Concepts and Objectives; File S1: Fidelity Observation Tool for Im-plementation of Teens CAN.
