# Supplementary material for: Building Food Literacy in Adolescence: A Pilot Study of the Teens CAN Curriculum
Source: Nutrients. 2026 Apr 30;18(9):1434. doi: 10.3390/nu18091434 (PMC13164871; doi:10.3390/nu18091434)
Supplement: Supplementary file 1 [file nutrients-18-01434-s001.zip › nutrients-4190994-supplementary.pdf]

Supplementary Table S1: Teens CAN Learning Concepts and Objectives [27].

**Lesson**	**Learning Concepts**	**Learning Objective (Youth will be able to…)**
Agriculture 1	Food supply chain	Organize components of the food supply chain
Agriculture 2	Agricultural systems	Compare and contrast various agricultural systems
Agriculture 3	Agroecology<br>Technology	Assemble a timeline of the various movements and advancements that have shaped the food system today
Agriculture 4	Food availability<br>Food access	Investigate deficits that exist in some food systems and ways to improve those deficits
Nutrition 1	MyPlate<br>Food groups	Evaluate why consuming a variety of foods is needed to help an individual meet their daily food group recommendations
Nutrition 2	Macronutrients<br>Micronutrients	Investigate which foods are good sources of different macronutrients and micronutrients utilizing the information provided on a Nutrition Facts Label
Nutrition 3	Nutrients of concern for underconsumption	Evaluate why consuming a variety of foods is needed to meet recommendations for nutrients of concern
Nutrition 4	Nutrition in media	Analyze nutrition claims in the media and critique misleading information
Cooking 1	Food safety	Identify and avoid potential food safety hazards
Cooking 2	Cooking equipment<br>Cooking techniques	Demonstrate proper utilization of various cooking techniques and equipment
Cooking 3	Seasonality<br>Budgeting	Plan meal options that incorporate budgetary needs and seasonal produce
Cooking 4	Recipe scaling<br>Serving and portion sizing	Plan a meal and calculate the cost per serving of that meal

Observation Tool for Implementation of Teens CAN

Observer___________________ **Educator**____________________ **Date**_____________

Activity A1 – Farm to Market Marathon

Program Fidelity

Curriculum Session	Program Fidelity	Notes
**Opening Questions**<br><br>Time started:<br>Time ended:	Asked opening questions<br>❑ Did not do<br>❑ Partially delivered according to curriculum<br>❑ Fully delivered according to curriculum	
**Procedure (Experiencing)**<br><br>Time started:<br>Time ended:	Conducted exploration of the food supply chain<br>❑ Did not do<br>❑ Partially delivered according to curriculum<br>❑ Fully delivered according to curriculum	
**Sharing, Processing and Generalizing**<br><br>Time started:<br>Time ended:	Youth shared and discussed their findings related to the food supply chain and what they learned<br>❑ Did not do<br>❑ Partially delivered according to curriculum<br>❑ Fully delivered according to curriculum<br>Facilitator followed up with prompts<br>❑ Did not do<br>❑ Partially delivered according to curriculum<br>❑ Fully delivered according to curriculum	
**Concept or Term Discovery/Introduction**<br><br>Time started:<br>Time ended:	Concepts and terms such as food supply chain, consumer, and producer were discovered by the youth or introduced by the facilitator<br>❑ Did not do<br>❑ Partially delivered according to curriculum<br>❑ Fully delivered according to curriculum	
**Terms**	❑ Food Supply Chain<br>❑ Producer<br>❑ Consumer<br>❑ Agriculture	❑ Direct Marketing<br>❑ Farmers Market<br>❑ Food Miles

Observation Tool for Implementation of Teens CAN

Program Quality

Questioning Strategies

Ratio Open-Ended : Closed Questions	
Number of Open	Number of Closed
**Examples of Questions Asked**	
**1**	
**2**	
**3**	
**4**	
**5**	
**6**	

Evidence of open-ended questioning

Percentage of open –ended questions:

- ❑ Between 0 and 24%
- ❑ Between 25- 49%
- ❑ Between 50- 74%
- ❑ Between 75-100%

Level of youth engagement

- ❑ None of the youth are interested and engaged
- ❑ About 25% of youth are interested and engaged
- ❑ About 50% of youth are interested and engaged
- ❑ About 75% of youth are interested and engaged
- ❑ 100% of the youth are interested and engaged

Level of youth engagement

- ❑ Overall, youth look bored and/or preoccupied
- ❑ Overall, youth look attentive, but silent
- ❑ Overall, youth look attentive and engaged in discussion

Level of youth participation compared to leader participation

- ❑ Youth talked less than 25% of the time (teacher talked 75% or more)
- ❑ Youth talked about 50% of the time (teacher 50%)
- ❑ Youth talked more than 75% of the time (teacher 25% or less)

Concept discovery/introduction

- ❑ No concepts were discovered or introduced during the session
- ❑ Concepts were not discovered or discussed by youth, but partially introduced by the teacher
- ❑ Concepts were discovered and/or fully discussed by youth or teacher

Observation Tool for Implementation of Teens CAN

Observer___________________ **Educator**____________________ **Date**_____________

Activity A2 – Exploring Agricultural Systems

Program Fidelity

Curriculum Session	Program Fidelity	Notes
**Opening Questions**<br><br>Time started:<br>Time ended:	Asked opening questions<br>❑ Did not do<br>❑ Partially delivered according to curriculum<br>❑ Fully delivered according to curriculum	
**Procedure (Experiencing)**<br><br>Time started:<br>Time ended:	Conducted exploration of various agricultural systems<br>❑ Did not do<br>❑ Partially delivered according to curriculum<br>❑ Fully delivered according to curriculum	
**Sharing, Processing and Generalizing**<br><br>Time started:<br>Time ended:	Youth shared and discussed their assigned agricultural system and what they learned<br>❑ Did not do<br>❑ Partially delivered according to curriculum<br>❑ Fully delivered according to curriculum<br>Facilitator followed up with prompts<br>❑ Did not do<br>❑ Partially delivered according to curriculum<br>❑ Fully delivered according to curriculum	
**Concept or Term Discovery/Introduction**<br><br>Time started:<br>Time ended:	Concepts and terms such as aquaponics, school gardens, and natural resources were discovered by the youth or introduced by the facilitator<br>❑ Did not do<br>❑ Partially delivered according to curriculum<br>❑ Fully delivered according to curriculum	
**Terms**	❑ Agricultural Systems<br>❑ Agroclimate<br>❑ Aquaponics<br>❑ Conventional Corn Farming<br>❑ Hopi Tribe	❑ Hydroponics<br>❑ Natural Resources<br>❑ School Gardens<br>❑ Vertical Farming

Observation Tool for Implementation of Teens CAN

Program Quality

Questioning Strategies

Ratio Open-Ended : Closed Questions	
Number of Open	Number of Closed

Examples of Questions Asked	
**1**	
**2**	
**3**	
**4**	
**5**	
**6**	

Evidence of open-ended questioning
Percentage of open –ended questions:

- ❑ Between 0 and 24%
- ❑ Between 25- 49%
- ❑ Between 50- 74%
- ❑ Between 75-100%

Level of youth engagement (1)

- ❑ None of the youth are interested and engaged
- ❑ About 25% of youth are interested and engaged
- ❑ About 50% of youth are interested and engaged
- ❑ About 75% of youth are interested and engaged
- ❑ 100% of the youth are interested and engaged

Level of youth engagement (2)

- ❑ Overall, youth look bored and/or preoccupied
- ❑ Overall, youth look attentive, but silent
- ❑ Overall, youth look attentive and engaged in discussion

Level of youth participation compared to leader participation

- ❑ Youth talked less than 25% of the time (teacher talked 75% or more)
- ❑ Youth talked about 50% of the time (teacher 50%)
- ❑ Youth talked more than 75% of the time (teacher 25% or less)

Concept discovery/introduction

- ❑ No concepts were discovered or introduced during the session
- ❑ Concepts were not discovered or discussed by youth, but partially introduced by the teacher
- ❑ Concepts were discovered and/or fully discussed by youth or teacher

Observation Tool for Implementation of Teens CAN

Observer___________________ Educator____________________ Date_____________

Activity A3 – Innovating Agriculture

Program Fidelity

Curriculum Session	Program Fidelity	Notes
**Opening Questions**<br><br>Time started:<br>Time ended:	Asked opening questions<br>❑ Did not do<br>❑ Partially delivered according to curriculum<br>❑ Fully delivered according to curriculum	
**Procedure (Experiencing)**<br><br>Time started:<br>Time ended:	Conducted exploration of various agricultural innovations<br>❑ Did not do<br>❑ Partially delivered according to curriculum<br>❑ Fully delivered according to curriculum	
**Sharing, Processing and Generalizing**<br><br>Time started:<br>Time ended:	Youth shared and discussed their agricultural innovation and what they learned<br>❑ Did not do<br>❑ Partially delivered according to curriculum<br>❑ Fully delivered according to curriculum<br>Facilitator followed up with prompts<br>❑ Did not do<br>❑ Partially delivered according to curriculum<br>❑ Fully delivered according to curriculum	
**Concept or Term Discovery/Introduction**<br><br>Time started:<br>Time ended:	Concepts and terms such as agroecology, GMOs, and nitrogen fixation, were discovered by the youth or introduced by the facilitator<br>❑ Did not do<br>❑ Partially delivered according to curriculum<br>❑ Fully delivered according to curriculum	
**Terms**	❑ Agroecology<br>❑ Drought<br>❑ Genetically Modified Organisms (GMOs)<br>❑ Global Positioning System (GPS)<br>❑ Nitrogen Fixation<br>❑ Pesticide<br>❑ Pesticide Resistance<br>❑ Soil Health<br>❑ Urban Sprawl	

Observation Tool for Implementation of Teens CAN

Program Quality

Questioning Strategies

Ratio Open-Ended : Closed Questions	
Number of Open	Number of Closed
**Examples of Questions Asked**	
**1**	
**2**	
**3**	
**4**	
**5**	
**6**	

Evidence of open-ended questioning

Percentage of open –ended questions:

- ❑ Between 0 and 24%
- ❑ Between 25- 49%
- ❑ Between 50- 74%
- ❑ Between 75-100%

Level of youth engagement (1)

- ❑ None of the youth are interested and engaged
- ❑ About 25% of youth are interested and engaged
- ❑ About 50% of youth are interested and engaged
- ❑ About 75% of youth are interested and engaged
- ❑ 100% of the youth are interested and engaged

Level of youth engagement (2)

- ❑ Overall, youth look bored and/or preoccupied
- ❑ Overall, youth look attentive, but silent
- ❑ Overall, youth look attentive and engaged in discussion

Level of youth participation compared to leader participation

- ❑ Youth talked less than 25% of the time (teacher talked 75% or more)
- ❑ Youth talked about 50% of the time (teacher 50%)
- ❑ Youth talked more than 75% of the time (teacher 25% or less)

Concept discovery/introduction

- ❑ No concepts were discovered or introduced during the session
- ❑ Concepts were not discovered or discussed by youth, but partially introduced by the teacher
- ❑ Concepts were discovered and/or fully discussed by youth or teacher

Observation Tool for Implementation of Teens CAN

Observer___________________ Educator____________________ Date_____________

Activity A4 – Food Desert Overhaul

Program Fidelity

Curriculum Session	Program Fidelity	Notes
**Opening Questions**<br><br>Time started:<br>Time ended:	Asked opening questions<br>❑ Did not do<br>❑ Partially delivered according to curriculum<br>❑ Fully delivered according to curriculum	
**Procedure (Experiencing)**<br><br>Time started:<br>Time ended:	Conducted exploration of food deserts and potential remedies<br>❑ Did not do<br>❑ Partially delivered according to curriculum<br>❑ Fully delivered according to curriculum	
**Sharing, Processing and Generalizing**<br><br>Time started:<br>Time ended:	Youth shared and discussed their renovated neighborhoods and what they learned<br>❑ Did not do<br>❑ Partially delivered according to curriculum<br>❑ Fully delivered according to curriculum<br>Facilitator followed up with prompts<br>❑ Did not do<br>❑ Partially delivered according to curriculum<br>❑ Fully delivered according to curriculum	
**Concept or Term Discovery/Introduction**<br><br>Time started:<br>Time ended:	Concepts and terms such as food desert, food access, food availability were discovered by the youth or introduced by the facilitator<br>❑ Did not do<br>❑ Partially delivered according to curriculum<br>❑ Fully delivered according to curriculum	
**Terms**	❑ Community Food Gardens<br>❑ Farmers Market<br>❑ Food Access<br>❑ Food Affordability<br>❑ Food Availability<br>❑ Food Deserts<br>❑ Food Swamps<br>❑ Health Zoning Laws	

Observation Tool for Implementation of Teens CAN

Program Quality

Questioning Strategies

Ratio Open-Ended : Closed Questions	
Number of Open	Number of Closed

Examples of Questions Asked	
**1**	
**2**	
**3**	
**4**	
**5**	
**6**	

Evidence of open-ended questioning

Percentage of open –ended questions:

- ❑ Between 0 and 24%
- ❑ Between 25- 49%
- ❑ Between 50- 74%
- ❑ Between 75-100%

Level of youth engagement (1)

- ❑ None of the youth are interested and engaged
- ❑ About 25% of youth are interested and engaged
- ❑ About 50% of youth are interested and engaged
- ❑ About 75% of youth are interested and engaged
- ❑ 100% of the youth are interested and engaged

Level of youth engagement (2)

- ❑ Overall, youth look bored and/or preoccupied
- ❑ Overall, youth look attentive, but silent
- ❑ Overall, youth look attentive and engaged in discussion

Level of youth participation compared to leader participation

- ❑ Youth talked less than 25% of the time (teacher talked 75% or more)
- ❑ Youth talked about 50% of the time (teacher 50%)
- ❑ Youth talked more than 75% of the time (teacher 25% or less)

Concept discovery/introduction

- ❑ No concepts were discovered or introduced during the session
- ❑ Concepts were not discovered or discussed by youth, but partially introduced by the teacher
- ❑ Concepts were discovered and/or fully discussed by youth or teacher

Observation Tool for Implementation of Teens CAN

Observer___________________ **Educator**____________________ **Date**_____________

Activity N1 – Your Plate Vs. MyPlate

Program Fidelity

Curriculum Session	Program Fidelity	Notes
**Opening Questions**<br><br>Time started:<br>Time ended:	Asked opening questions<br>❑ Did not do<br>❑ Partially delivered according to curriculum<br>❑ Fully delivered according to curriculum	
**Procedure (Experiencing)**<br><br>Time started:<br>Time ended:	Conducted exploration of food groups and MyPlate recommendations<br>❑ Did not do<br>❑ Partially delivered according to curriculum<br>❑ Fully delivered according to curriculum	
**Sharing, Processing and Generalizing**<br><br>Time started:<br>Time ended:	Youth shared and discussed their characters' eating patterns and what they learned<br>❑ Did not do<br>❑ Partially delivered according to curriculum<br>❑ Fully delivered according to curriculum<br>Facilitator followed up with prompts<br>❑ Did not do<br>❑ Partially delivered according to curriculum<br>❑ Fully delivered according to curriculum	
**Concept or Term Discovery/Introduction**<br><br>Time started:<br>Time ended:	Concepts and terms such as MyPlate, food groups, and activity level were discovered by the youth or introduced by the facilitator<br>❑ Did not do<br>❑ Partially delivered according to curriculum<br>❑ Fully delivered according to curriculum	
**Terms**	❑ Dairy<br>❑ Eating Pattern<br>❑ Food Groups<br>❑ Fruits<br>❑ Grains<br>❑ Moderate Activity Level<br>❑ MyPlate	❑ Oils<br>❑ Physical Activity Intensity Level<br>❑ Protein Foods<br>❑ Sedentary Activity Level<br>❑ Vegetables<br>❑ Vigorous Activity Level

Observation Tool for Implementation of Teens CAN

Program Quality

Questioning Strategies

Ratio Open-Ended : Closed Questions	
Number of Open	Number of Closed

Examples of Questions Asked	
**1**	
**2**	
**3**	
**4**	
**5**	
**6**	

Evidence of open-ended questioning

Percentage of open –ended questions:

- ❑ Between 0 and 24%
- ❑ Between 25- 49%
- ❑ Between 50- 74%
- ❑ Between 75-100%

Level of youth engagement (1)

- ❑ None of the youth are interested and engaged
- ❑ About 25% of youth are interested and engaged
- ❑ About 50% of youth are interested and engaged
- ❑ About 75% of youth are interested and engaged
- ❑ 100% of the youth are interested and engaged

Level of youth engagement (2)

- ❑ Overall, youth look bored and/or preoccupied
- ❑ Overall, youth look attentive, but silent
- ❑ Overall, youth look attentive and engaged in discussion

Level of youth participation compared to leader participation

- ❑ Youth talked less than 25% of the time (teacher talked 75% or more)
- ❑ Youth talked about 50% of the time (teacher 50%)
- ❑ Youth talked more than 75% of the time (teacher 25% or less)

Concept discovery/introduction

- ❑ No concepts were discovered or introduced during the session
- ❑ Concepts were not discovered or discussed by youth, but partially introduced by the teacher
- ❑ Concepts were discovered and/or fully discussed by youth or teacher

Observation Tool for Implementation of Teens CAN

Observer___________________ **Educator**____________________ **Date**_____________

Activity N2 – Read It to Eat It

Program Fidelity

Curriculum Session	Program Fidelity	Notes
**Opening Questions**<br><br>Time started:<br>Time ended:	Asked opening questions<br>❑ Did not do<br>❑ Partially delivered according to curriculum<br>❑ Fully delivered according to curriculum	
**Procedure (Experiencing)**<br><br>Time started:<br>Time ended:	Conducted exploration of nutrients and Nutrition Facts Labels<br>❑ Did not do<br>❑ Partially delivered according to curriculum<br>❑ Fully delivered according to curriculum	
**Sharing, Processing and Generalizing**<br><br>Time started:<br>Time ended:	Youth shared and discussed good sources of different nutrients and what they learned<br>❑ Did not do<br>❑ Partially delivered according to curriculum<br>❑ Fully delivered according to curriculum<br>Facilitator followed up with prompts<br>❑ Did not do<br>❑ Partially delivered according to curriculum<br>❑ Fully delivered according to curriculum	
**Concept or Term Discovery/Introduction**<br><br>Time started:<br>Time ended:	Concepts and terms such as macronutrients, micronutrients, and Nutrition Facts Labels were discovered by the youth or introduced by the facilitator<br>❑ Did not do<br>❑ Partially delivered according to curriculum<br>❑ Fully delivered according to curriculum	
**Terms**	❑ Carbohydrate<br>❑ Dietary Fiber<br>❑ Fat<br>❑ Fortification<br>❑ Macronutrient<br>❑ Micronutrient<br>❑ Minerals	❑ Nutrient<br>❑ Nutrition Facts Label<br>❑ Percent Daily Value<br>❑ Protein<br>❑ Serving size<br>❑ Vitamins

Observation Tool for Implementation of Teens CAN

Program Quality

Questioning Strategies

Ratio Open-Ended : Closed Questions		
Number of Open		Number of Closed
Examples of Questions Asked		
**1**		
**2**		
**3**		
**4**		
**5**		
**6**		

Evidence of open-ended questioning
Percentage of open –ended questions:
- ❑ Between 0 and 24%
- ❑ Between 25- 49%
- ❑ Between 50- 74%
- ❑ Between 75-100%

Level of youth engagement (1)
- ❑ None of the youth are interested and engaged
- ❑ About 25% of youth are interested and engaged
- ❑ About 50% of youth are interested and engaged
- ❑ About 75% of youth are interested and engaged
- ❑ 100% of the youth are interested and engaged

Level of youth engagement (2)
- ❑ Overall, youth look bored and/or preoccupied
- ❑ Overall, youth look attentive, but silent
- ❑ Overall, youth look attentive and engaged in discussion

Level of youth participation compared to leader participation
- ❑ Youth talked less than 25% of the time (teacher talked 75% or more)
- ❑ Youth talked about 50% of the time (teacher 50%)
- ❑ Youth talked more than 75% of the time (teacher 25% or less)

Concept discovery/introduction
- ❑ No concepts were discovered or introduced during the session
- ❑ Concepts were not discovered or discussed by youth, but partially introduced by the teacher
- ❑ Concepts were discovered and/or fully discussed by youth or teacher

Observation Tool for Implementation of Teens CAN

Observer____________________ Educator_____________________ Date_____________

Activity N3 – Nutrition for All

Program Fidelity

Curriculum Session	Program Fidelity	Notes
**Opening Questions**<br><br>Time started:<br>Time ended:	Asked opening questions<br>❑ Did not do<br>❑ Partially delivered according to curriculum<br>❑ Fully delivered according to curriculum	
**Procedure (Experiencing)**<br><br>Time started:<br>Time ended:	Conducted exploration of nutrients of concern and nutrient recommendations<br>❑ Did not do<br>❑ Partially delivered according to curriculum<br>❑ Fully delivered according to curriculum	
**Sharing, Processing and Generalizing**<br><br>Time started:<br>Time ended:	Youth shared and discussed recommendations for nutrients of concern and what they learned<br>❑ Did not do<br>❑ Partially delivered according to curriculum<br>❑ Fully delivered according to curriculum<br>Facilitator followed up with prompts<br>❑ Did not do<br>❑ Partially delivered according to curriculum<br>❑ Fully delivered according to curriculum	
**Concept or Term Discovery/Introduction**<br><br>Time started:<br>Time ended:	Concepts and terms such as nutrients of concern, DRIs, and nutrient density were discovered by the youth or introduced by the facilitator<br>❑ Did not do<br>❑ Partially delivered according to curriculum<br>❑ Fully delivered according to curriculum	
**Terms**	❑ Calcium<br>❑ Dietary Fiber<br>❑ Dietary Reference Intakes (DRIs)<br>❑ Fortification<br>❑ Iron<br>❑ Macronutrient<br>❑ Metabolism	❑ Micronutrient<br>❑ Nutrient<br>❑ Nutrient Composition<br>❑ Nutrient-Dense<br>❑ Nutrient of Concern<br>❑ Potassium<br>❑ Supplement<br>❑ Vitamin D

Observation Tool for Implementation of Teens CAN

Program Quality

Questioning Strategies

Ratio Open-Ended : Closed Questions	
Number of Open	Number of Closed

Examples of Questions Asked	
**1**	
**2**	
**3**	
**4**	
**5**	
**6**	

Evidence of open-ended questioning

Percentage of open –ended questions:

- ❑ Between 0 and 24%
- ❑ Between 25- 49%
- ❑ Between 50- 74%
- ❑ Between 75-100%

Level of youth engagement (1)

- ❑ None of the youth are interested and engaged
- ❑ About 25% of youth are interested and engaged
- ❑ About 50% of youth are interested and engaged
- ❑ About 75% of youth are interested and engaged
- ❑ 100% of the youth are interested and engaged

Level of youth engagement (2)

- ❑ Overall, youth look bored and/or preoccupied
- ❑ Overall, youth look attentive, but silent
- ❑ Overall, youth look attentive and engaged in discussion

Level of youth participation compared to leader participation

- ❑ Youth talked less than 25% of the time (teacher talked 75% or more)
- ❑ Youth talked about 50% of the time (teacher 50%)
- ❑ Youth talked more than 75% of the time (teacher 25% or less)

Concept discovery/introduction

- ❑ No concepts were discovered or introduced during the session
- ❑ Concepts were not discovered or discussed by youth, but partially introduced by the teacher
- ❑ Concepts were discovered and/or fully discussed by youth or teacher

Observation Tool for Implementation of Teens CAN

Observer___________________ **Educator**____________________ **Date**_____________

Activity N4 – Fact or Fiction

Program Fidelity

Curriculum Session	Program Fidelity	Notes
**Opening Questions**<br><br>Time started:<br>Time ended:	Asked opening questions<br>❑ Did not do<br>❑ Partially delivered according to curriculum<br>❑ Fully delivered according to curriculum	
**Procedure (Experiencing)**<br><br>Time started:<br>Time ended:	Conducted exploration of marketing strategies and nutrition media<br>❑ Did not do<br>❑ Partially delivered according to curriculum<br>❑ Fully delivered according to curriculum	
**Sharing, Processing and Generalizing**<br><br>Time started:<br>Time ended:	Youth shared and discussed nutrition advertisements and what they learned<br>❑ Did not do<br>❑ Partially delivered according to curriculum<br>❑ Fully delivered according to curriculum<br>Facilitator followed up with prompts<br>❑ Did not do<br>❑ Partially delivered according to curriculum<br>❑ Fully delivered according to curriculum	
**Concept or Term Discovery/Introduction**<br><br>Time started:<br>Time ended:	Concepts and terms such as marketing strategies and reliable resources were discovered by the youth or introduced by the facilitator<br>❑ Did not do<br>❑ Partially delivered according to curriculum<br>❑ Fully delivered according to curriculum	
**Terms**	❑ Advertisement<br>❑ Appeal<br>❑ Branding<br>❑ Consumer<br>❑ Marketing strategies<br>❑ Promotion<br>❑ Publicity<br>❑ Reliable Resources	

Observation Tool for Implementation of Teens CAN

Program Quality

Questioning Strategies

Ratio Open-Ended : Closed Questions	
Number of Open	Number of Closed

Examples of Questions Asked	
**1**	
**2**	
**3**	
**4**	
**5**	
**6**	

Evidence of open-ended questioning

Percentage of open –ended questions:

- ❑ Between 0 and 24%
- ❑ Between 25- 49%
- ❑ Between 50- 74%
- ❑ Between 75-100%

Level of youth engagement (1)

- ❑ None of the youth are interested and engaged
- ❑ About 25% of youth are interested and engaged
- ❑ About 50% of youth are interested and engaged
- ❑ About 75% of youth are interested and engaged
- ❑ 100% of the youth are interested and engaged

Level of youth engagement (2)

- ❑ Overall, youth look bored and/or preoccupied
- ❑ Overall, youth look attentive, but silent
- ❑ Overall, youth look attentive and engaged in discussion

Level of youth participation compared to leader participation

- ❑ Youth talked less than 25% of the time (teacher talked 75% or more)
- ❑ Youth talked about 50% of the time (teacher 50%)
- ❑ Youth talked more than 75% of the time (teacher 25% or less)

Concept discovery/introduction

- ❑ No concepts were discovered or introduced during the session
- ❑ Concepts were not discovered or discussed by youth, but partially introduced by the teacher
- ❑ Concepts were discovered and/or fully discussed by youth or teacher

Observation Tool for Implementation of Teens CAN

Observer___________________ **Educator**____________________ **Date**_____________

Activity C1 – Fact or Fiction

Program Fidelity

Curriculum Session	Program Fidelity	Notes
**Opening Questions**<br><br>Time started:<br>Time ended:	Asked opening questions<br>❑ Did not do<br>❑ Partially delivered according to curriculum<br>❑ Fully delivered according to curriculum	
**Procedure (Experiencing)**<br><br>Time started:<br>Time ended:	Conducted exploration of food safety practices<br>❑ Did not do<br>❑ Partially delivered according to curriculum<br>❑ Fully delivered according to curriculum	
**Sharing, Processing and Generalizing**<br><br>Time started:<br>Time ended:	Youth shared and discussed food safety practices and what they learned<br>❑ Did not do<br>❑ Partially delivered according to curriculum<br>❑ Fully delivered according to curriculum<br>Facilitator followed up with prompts<br>❑ Did not do<br>❑ Partially delivered according to curriculum<br>❑ Fully delivered according to curriculum	
**Concept or Term Discovery/Introduction**<br><br>Time started:<br>Time ended:	Concepts and terms such as cross-contamination, food safety, and foodborne illness were discovered by the youth or introduced by the facilitator<br>❑ Did not do<br>❑ Partially delivered according to curriculum<br>❑ Fully delivered according to curriculum	
**Terms**	❑ Bacteria<br>❑ Cross-Contamination<br>❑ Danger Zone<br>❑ Foodborne Illness<br>❑ Food Safety	

Program Quality

Questioning Strategies

Ratio Open-Ended : Closed Questions	
Number of Open	Number of Closed
Examples of Questions Asked	
**1**	
**2**	
**3**	
**4**	
**5**	
**6**	

Evidence of open-ended questioning

Percentage of open –ended questions:

- ❑ Between 0 and 24%
- ❑ Between 25- 49%
- ❑ Between 50- 74%
- ❑ Between 75-100%

Level of youth engagement (1)

- ❑ None of the youth are interested and engaged
- ❑ About 25% of youth are interested and engaged
- ❑ About 50% of youth are interested and engaged
- ❑ About 75% of youth are interested and engaged
- ❑ 100% of the youth are interested and engaged

Level of youth engagement (2)

- ❑ Overall, youth look bored and/or preoccupied
- ❑ Overall, youth look attentive, but silent
- ❑ Overall, youth look attentive and engaged in discussion

Level of youth participation compared to leader participation

- ❑ Youth talked less than 25% of the time (teacher talked 75% or more)
- ❑ Youth talked about 50% of the time (teacher 50%)
- ❑ Youth talked more than 75% of the time (teacher 25% or less)

Concept discovery/introduction

- ❑ No concepts were discovered or introduced during the session
- ❑ Concepts were not discovered or discussed by youth, but partially introduced by the teacher
- ❑ Concepts were discovered and/or fully discussed by youth or teacher

Observation Tool for Implementation of Teens CAN

Observer____________________ **Educator**____________________ **Date**_____________

Activity C2 – Chop, Chop, Dice

Program Fidelity

Curriculum Session	Program Fidelity	Notes
**Opening Questions**<br><br>Time started:<br>Time ended:	Asked opening questions<br>❑ Did not do<br>❑ Partially delivered according to curriculum<br>❑ Fully delivered according to curriculum	
**Procedure (Experiencing)**<br><br>Time started:<br>Time ended:	Conducted exploration of cooking techniques and equipment<br>❑ Did not do<br>❑ Partially delivered according to curriculum<br>❑ Fully delivered according to curriculum	
**Sharing, Processing and Generalizing**<br><br>Time started:<br>Time ended:	Youth shared and discussed cooking skills and safety and what they learned<br>❑ Did not do<br>❑ Partially delivered according to curriculum<br>❑ Fully delivered according to curriculum<br>Facilitator followed up with prompts<br>❑ Did not do<br>❑ Partially delivered according to curriculum<br>❑ Fully delivered according to curriculum	
**Concept or Term Discovery/Introduction**<br><br>Time started:<br>Time ended:	Concepts and terms such as knife safety, dicing, cooking techniques were discovered by the youth or introduced by the facilitator<br>❑ Did not do<br>❑ Partially delivered according to curriculum<br>❑ Fully delivered according to curriculum	
**Terms**	❑ Boiling<br>❑ Chopping<br>❑ Cooking Techniques<br>❑ Dice<br>❑ Knife Safety<br>❑ Mincing<br>❑ Mise en place<br>❑ Sauteing<br>❑ Slicing<br>❑ The Draw	

Observation Tool for Implementation of Teens CAN

Program Quality

Questioning Strategies

Ratio Open-Ended : Closed Questions	
Number of Open	Number of Closed

Examples of Questions Asked	
**1**	
**2**	
**3**	
**4**	
**5**	
**6**	

Evidence of open-ended questioning
Percentage of open –ended questions:

- ❑ Between 0 and 24%
- ❑ Between 25- 49%
- ❑ Between 50- 74%
- ❑ Between 75-100%

Level of youth engagement (1)

- ❑ None of the youth are interested and engaged
- ❑ About 25% of youth are interested and engaged
- ❑ About 50% of youth are interested and engaged
- ❑ About 75% of youth are interested and engaged
- ❑ 100% of the youth are interested and engaged

Level of youth engagement (2)

- ❑ Overall, youth look bored and/or preoccupied
- ❑ Overall, youth look attentive, but silent
- ❑ Overall, youth look attentive and engaged in discussion

Level of youth participation compared to leader participation

- ❑ Youth talked less than 25% of the time (teacher talked 75% or more)
- ❑ Youth talked about 50% of the time (teacher 50%)
- ❑ Youth talked more than 75% of the time (teacher 25% or less)

Concept discovery/introduction

- ❑ No concepts were discovered or introduced during the session
- ❑ Concepts were not discovered or discussed by youth, but partially introduced by the teacher
- ❑ Concepts were discovered and/or fully discussed by youth or teacher

Observation Tool for Implementation of Teens CAN

Observer___________________ Educator____________________ Date_____________

Activity C3 – Shopping with the Seasons

Program Fidelity

Curriculum Session	Program Fidelity	Notes
**Opening Questions**<br><br>Time started:<br>Time ended:	Asked opening questions<br>❑ Did not do<br>❑ Partially delivered according to curriculum<br>❑ Fully delivered according to curriculum	
**Procedure (Experiencing)**<br><br>Time started:<br>Time ended:	Conducted exploration of seasonal grocery shopping<br>❑ Did not do<br>❑ Partially delivered according to curriculum<br>❑ Fully delivered according to curriculum	
**Sharing, Processing and Generalizing**<br><br>Time started:<br>Time ended:	Youth shared and discussed budgets and shopping lists and what they learned<br>❑ Did not do<br>❑ Partially delivered according to curriculum<br>❑ Fully delivered according to curriculum<br>Facilitator followed up with prompts<br>❑ Did not do<br>❑ Partially delivered according to curriculum<br>❑ Fully delivered according to curriculum	
**Concept or Term Discovery/Introduction**<br><br>Time started:<br>Time ended:	Concepts and terms such as budget, seasonality, and meal planning were discovered by the youth or introduced by the facilitator<br>❑ Did not do<br>❑ Partially delivered according to curriculum<br>❑ Fully delivered according to curriculum	
**Terms**	❑ Budget<br>❑ Buying in Bulk<br>❑ Conventional Produce<br>❑ Economical Grocery Shopping<br>❑ Meal Planning<br>❑ Organic Produce<br>❑ Seasonal Produce	

Observation Tool for Implementation of Teens CAN

Program Quality

Questioning Strategies

Ratio Open-Ended : Closed Questions	
Number of Open	Number of Closed
Examples of Questions Asked	
**1**	
**2**	
**3**	
**4**	
**5**	
**6**	

Evidence of open-ended questioning

Percentage of open –ended questions:

- ❑ Between 0 and 24%
- ❑ Between 25- 49%
- ❑ Between 50- 74%
- ❑ Between 75-100%

Level of youth engagement (1)

- ❑ None of the youth are interested and engaged
- ❑ About 25% of youth are interested and engaged
- ❑ About 50% of youth are interested and engaged
- ❑ About 75% of youth are interested and engaged
- ❑ 100% of the youth are interested and engaged

Level of youth engagement (2)

- ❑ Overall, youth look bored and/or preoccupied
- ❑ Overall, youth look attentive, but silent
- ❑ Overall, youth look attentive and engaged in discussion

Level of youth participation compared to leader participation

- ❑ Youth talked less than 25% of the time (teacher talked 75% or more)
- ❑ Youth talked about 50% of the time (teacher 50%)
- ❑ Youth talked more than 75% of the time (teacher 25% or less)

Concept discovery/introduction

- ❑ No concepts were discovered or introduced during the session
- ❑ Concepts were not discovered or discussed by youth, but partially introduced by the teacher
- ❑ Concepts were discovered and/or fully discussed by youth or teacher

Observation Tool for Implementation of Teens CAN

Observer___________________ **Educator**____________________ **Date**_____________

Activity C4 – Food Fractioning

Program Fidelity

Curriculum Session	Program Fidelity	Notes
**Opening Questions**<br><br>Time started:<br>Time ended:	Asked opening questions<br>❑ Did not do<br>❑ Partially delivered according to curriculum<br>❑ Fully delivered according to curriculum	
**Procedure (Experiencing)**<br><br>Time started:<br>Time ended:	Conducted exploration of serving versus portion sizes<br>❑ Did not do<br>❑ Partially delivered according to curriculum<br>❑ Fully delivered according to curriculum	
**Sharing, Processing and Generalizing**<br><br>Time started:<br>Time ended:	Youth shared and discussed prices per portion and what they learned<br>❑ Did not do<br>❑ Partially delivered according to curriculum<br>❑ Fully delivered according to curriculum<br>Facilitator followed up with prompts<br>❑ Did not do<br>❑ Partially delivered according to curriculum<br>❑ Fully delivered according to curriculum	
**Concept or Term Discovery/Introduction**<br><br>Time started:<br>Time ended:	Concepts and terms such as portion size, serving size, and recipe scaling were discovered by the youth or introduced by the facilitator<br>❑ Did not do<br>❑ Partially delivered according to curriculum<br>❑ Fully delivered according to curriculum	
**Terms**	❑ Nutrition Facts Label<br>❑ Serving Size<br>❑ Portion Size<br>❑ Recipe Scaling	

Observation Tool for Implementation of Teens CAN

Program Quality

Questioning Strategies

Ratio Open-Ended : Closed Questions		
Number of Open		Number of Closed
Examples of Questions Asked		
**1**		
**2**		
**3**		
**4**		
**5**		
**6**		

Evidence of open-ended questioning

Percentage of open –ended questions:

- ❑ Between 0 and 24%
- ❑ Between 25- 49%
- ❑ Between 50- 74%
- ❑ Between 75-100%

Level of youth engagement (1)

- ❑ None of the youth are interested and engaged
- ❑ About 25% of youth are interested and engaged
- ❑ About 50% of youth are interested and engaged
- ❑ About 75% of youth are interested and engaged
- ❑ 100% of the youth are interested and engaged

Level of youth engagement (2)

- ❑ Overall, youth look bored and/or preoccupied
- ❑ Overall, youth look attentive, but silent
- ❑ Overall, youth look attentive and engaged in discussion

Level of youth participation compared to leader participation

- ❑ Youth talked less than 25% of the time (teacher talked 75% or more)
- ❑ Youth talked about 50% of the time (teacher 50%)
- ❑ Youth talked more than 75% of the time (teacher 25% or less)

Concept discovery/introduction

- ❑ No concepts were discovered or introduced during the session
- ❑ Concepts were not discovered or discussed by youth, but partially introduced by the teacher
- ❑ Concepts were discovered and/or fully discussed by youth or teacher
