# Supplementary material for: Building Food Literacy in Adolescence: A Pilot Study of the Teens CAN Curriculum
Source: Nutrients. 2026 Apr 30;18(9):1434. doi: 10.3390/nu18091434 (PMC13164871; doi:10.3390/nu18091434)
Supplement: Supplementary file 1 [file nutrients-18-01434-s001.zip › Minimal Dataset/Teens CAN Main Dataset Codebook and Data Structure Guide.pdf]

Teens CAN Main Dataset Codebook and Data Structure Guide

Study Design: Pre–Post quasi-experimental design with comparison group
Unit of Analysis: Individual participant
Data File Format: Excel (.xlsx)
File Name: Teens CAN Coded Data.xlsx
Coding Software Used: R
Groups:

- Intervention = 1
- Comparison/Control = 0

Time Points:

- Pre-intervention (baseline)
- Post-intervention

Primary Outcomes:

- Nutrition Knowledge (NK)
- Motivation (Mot)
- Fruit intake
- Vegetable intake
- Sugar-sweetened beverage intake (SSB)
- Composite diet score

With the excel each row represents one participant with participants identified using a unique ID. Each column represents a variable. Pre = Baseline; Post = Follow-up; Change = Post minus Pre. Higher scores indicate greater levels of the construct unless otherwise specified.

Variable Name	Type	Description	Interpretation
Group	Categorical	Study group assignment (0 = Control, 1 = Intervention)	0/1 coding
NKPre	Continuous	Nutrition Knowledge score at baseline	Higher = greater knowledge
NKPost	Continuous	Nutrition Knowledge score at follow-up	Higher = greater knowledge
NK_change	Continuous	Change in Nutrition Knowledge (Post – Pre)	Positive = improvement in knowledge from pre to post
MotPre	Continuous	Motivation score at baseline	Higher = greater motivation
MotPost	Continuous	Motivation score at follow-up	Higher = greater motivation
Mot_change	Continuous	Change in Motivation (Post – Pre)	Positive = improvement in motivation from pre to post
FruitTotalPre	Continuous	Total fruit intake at baseline	Higher = greater fruit intake
FruitTotalPost	Continuous	Total fruit intake at follow-up	Higher = greater fruit intake

FruitTotal_change	Continuous	Change in fruit intake (Post – Pre)	Positive = increased fruit intake from pre to post
VegTotalPre	Continuous	Total vegetable intake at baseline	Higher = greater vegetable intake
VegTotalPost	Continuous	Total vegetable intake at follow-up	Higher = greater vegetable intake
VegTotal_change	Continuous	Change in vegetable intake (Post – Pre)	Positive = increased vegetable intake from pre to post
FVTotalPre	Continuous	Total fruit + vegetable intake at baseline	Higher = greater total fruit and vegetable intake
FVTotalPost	Continuous	Total fruit + vegetable intake at follow-up	Higher = greater total fruit and vegetable intake
FVTotal_change	Continuous	Change in total fruit + vegetable intake (Post – Pre)	Positive = improvement in total fruit and vegetable intake from pre to post
SSBTotalPre	Continuous	Sugar-sweetened beverage intake at baseline	Higher = greater SSB intake
SSBTotalPost	Continuous	Sugar-sweetened beverage intake at follow-up	Higher = greater SSB intake
SSBTotal_change	Continuous	Change in SSB intake (Post – Pre)	Negative = a decrease in SSB intake from pre to post
DietPre	Continuous	Composite diet score (Fruit + Veg – SSB) at baseline	Higher = Greater composite diet quality
DietPost	Continuous	Composite diet score at follow-up	Higher = Greater composite diet quality
DietChange	Continuous	Change in composite diet score (Post – Pre)	Positive = improved composite diet quality from pre to post

Composite diet score code:
DietPre = FruitTotalPre + VegTotalPre - SSBTotalPre
DietPost = FruitTotalPost + VegTotalPost - SSBTotalPost
DietChange = DietPost – DietPre
Analysis:

- Independent samples t-tests (group differences)
- Change score analysis (Post – Pre)
- ANCOVA models controlling for baseline values

Missing Data: Missing values coded as: NA

- Sample sizes may vary by outcome variable

Teens CAN Demographic Dataset Codebook and Data Structure Guide

Study Design: Pre–Post quasi-experimental design with comparison group
Unit of Analysis: Individual participant
Data File Format: Excel (.xlsx)
File Name: Teens CAN Demographics
File contains:
Groups:

- Intervention = 1
- Comparison/Control = 0

Primary Outcomes:

- Race/Ethnicity (White, not of Hispanic Origin = 1; Hispanic = 2; Non-Hispanic Black = 3; Asian/Pacific Islander = 4; Middle Eastern = 5; Multi-Racial/Other = 6)
- Gender Identity (Male = 1; Female = 2; Self-identify = 3)
- Age: Reported in years
- BMI: Reported in kg/m^2
- Tobacco exposure (No = 1; Yes = 2)

Analysis:
Demographic balance assessed using Fisher's Exact Test (appropriate for small samples)

Missing Data: Missing values coded as: NA

- Sample sizes may vary by outcome variable
