# Supplementary material for: Building Food Literacy in Adolescence: A Pilot Study of the Teens CAN Curriculum
Source: Nutrients. 2026 Apr 30;18(9):1434. doi: 10.3390/nu18091434 (PMC13164871; doi:10.3390/nu18091434)
Supplement: Supplementary file 1 [file nutrients-18-01434-s001.zip › Minimal Dataset/Teens Can R Code.pdf]

```r
library(readxl)
TEENSCAN <- read_excel("~/Desktop/TEENSCAN.xlsx")
View(TEENSCAN)

library(dplyr)

# Knowledge -------------------------------------------------------------------------------
-----
TEENSCAN %>%
  group_by(Group) %>%
  summarise(
    mean_NKPre = mean(NKPre, na.rm = TRUE),
    sd_NKpre   = sd(NKPre, na.rm = TRUE),
    n          = sum(!is.na(NKPre))
  )

TEENSCAN %>%
  group_by(Group) %>%
  summarise(
    mean_NKPost2 = mean(NKPost, na.rm = TRUE),
    sd_NKPost2   = sd(NKPost, na.rm = TRUE),
    n          = sum(!is.na(NKPost))
  )

t.test(NKPre~ Group, data = TEENSCAN)
t.test(NKPost ~ Group, data = TEENSCAN)

TEENSCAN <- TEENSCAN %>%
  mutate(NK_change = NKPost - NKPre)

TEENSCAN %>%
  group_by(Group) %>%
  summarise(
    mean_change = mean(NK_change, na.rm = TRUE),
    sd_change   = sd(NK_change, na.rm = TRUE),
    n           = sum(!is.na(NK_change))
  )

t.test(NK_change ~ Group, data = TEENSCAN)


# Motivators ------------------------------------------------------------------------------
----------



TEENSCAN %>%
  group_by(Group) %>%
  summarise(
    mean_Mot = mean(MotPre, na.rm = TRUE),
    sd_Mot   = sd(MotPre, na.rm = TRUE),
    n          = sum(!is.na(MotPre))
  )


TEENSCAN %>%
  group_by(Group) %>%
  summarise(
    mean_Mot2 = mean(MotPost, na.rm = TRUE),
    sd_Mot2   = sd(MotPost, na.rm = TRUE),
    n          = sum(!is.na(MotPost))
  )

t.test(MotPre~ Group, data = TEENSCAN)
t.test(MotPost ~ Group, data = TEENSCAN)
```

```
TEENSCAN <- TEENSCAN %>%
  mutate(Mot_change = MotPost - MotPre)

TEENSCAN %>%
  group_by(Group) %>%
  summarise(
    Motmean_change = mean(Mot_change, na.rm = TRUE),
    Motsd_change   = sd(Mot_change, na.rm = TRUE),
    n            = sum(!is.na(Mot_change))
  )

t.test(Mot_change ~ Group, data = TEENSCAN)



# FVtot ------------------------------------------------------------------------------
----------

TEENSCAN %>%
  group_by(Group) %>%
  summarise(
    mean_FVT = mean(FVTotalPre, na.rm = TRUE),
    sd_FVT   = sd(FVTotalPre, na.rm = TRUE),
    n           = sum(!is.na(FVTotalPre))
  )

# FVtot
TEENSCAN %>%
  group_by(Group) %>%
  summarise(
    mean_FVT2 = mean(FVTotalPost, na.rm = TRUE),
    sd_FVT2   = sd(FVTotalPost, na.rm = TRUE),
    n           = sum(!is.na(FVTotalPost))
  )

t.test(FVTotalPre~ Group, data = TEENSCAN)
t.test(FVTotalPost ~ Group, data = TEENSCAN)

TEENSCAN <- TEENSCAN %>%
  mutate(FVTotal_change = FVTotalPost - FVTotalPre)

TEENSCAN %>%
  group_by(Group) %>%
  summarise(
    FVTmean_change = mean(FVTotal_change, na.rm = TRUE),
    FVTsd_change   = sd(FVTotal_change, na.rm = TRUE),
    n            = sum(!is.na(FVTotal_change))
  )

t.test(FVTotal_change ~ Group, data = TEENSCAN)


# Fruittot ---------------------------------------------------------------------------
----------

TEENSCAN %>%
  group_by(Group) %>%
  summarise(
    mean_Fruit = mean(FruitTotalPre, na.rm = TRUE),
    sd_Fruit   = sd(FruitTotalPre, na.rm = TRUE),
    n           = sum(!is.na(FruitTotalPre))
  )
```

```
# Fruittot
TEENSCAN %>%
  group_by(Group) %>%
  summarise(
    mean_Fruit2 = mean(FruitTotalPost, na.rm = TRUE),
    sd_Fruit2   = sd(FruitTotalPost, na.rm = TRUE),
    n          = sum(!is.na(FruitTotalPost))
  )

t.test(FruitTotalPre~ Group, data = TEENSCAN)
t.test(FruitTotalPost ~ Group, data = TEENSCAN)

TEENSCAN <- TEENSCAN %>%
  mutate(FruitTotal_change = FruitTotalPost - FruitTotalPre)

TEENSCAN %>%
  group_by(Group) %>%
  summarise(
    Fruitmean_change = mean(FruitTotal_change, na.rm = TRUE),
    Fruitsd_change   = sd(FruitTotal_change, na.rm = TRUE),
    n           = sum(!is.na(FruitTotal_change))
  )

t.test(FruitTotal_change ~ Group, data = TEENSCAN)
# Veg tot ----------------------------------------------------------------------------------------------
----------

TEENSCAN %>%
  group_by(Group) %>%
  summarise(
    mean_Veg = mean(VegTotalPre, na.rm = TRUE),
    sd_Veg   = sd(VegTotalPre, na.rm = TRUE),
    n          = sum(!is.na(VegTotalPre))
  )

TEENSCAN %>%
  group_by(Group) %>%
  summarise(
    mean_Veg2 = mean(VegTotalPost, na.rm = TRUE),
    sd_Veg2   = sd(VegTotalPost, na.rm = TRUE),
    n          = sum(!is.na(VegTotalPost))
  )

t.test(VegTotalPre~ Group, data = TEENSCAN)
t.test(VegTotalPost ~ Group, data = TEENSCAN)

TEENSCAN <- TEENSCAN %>%
  mutate(VegTotal_change = VegTotalPost - VegTotalPre)

TEENSCAN %>%
  group_by(Group) %>%
  summarise(
    Vegmean_change = mean(VegTotal_change, na.rm = TRUE),
    Vegsd_change   = sd(VegTotal_change, na.rm = TRUE),
    n           = sum(!is.na(VegTotal_change))
  )

t.test(VegTotal_change ~ Group, data = TEENSCAN)

# SSB tot ----------------------------------------------------------------------------------------------
----------

TEENSCAN %>%
  group_by(Group) %>%
```

```
  summarise(
    mean_SSB = mean(SSBTotalPre, na.rm = TRUE),
    sd_SSB   = sd(SSBTotalPre, na.rm = TRUE),
    n          = sum(!is.na(SSBTotalPre))
  )

TEENSCAN %>%
  group_by(Group) %>%
  summarise(
    mean_SSB2 = mean(SSBTotalPost, na.rm = TRUE),
    sd_SSB2   = sd(SSBTotalPost, na.rm = TRUE),
    n          = sum(!is.na(SSBTotalPost))
  )

t.test(SSBTotalPre~ Group, data = TEENSCAN)
t.test(SSBTotalPost ~ Group, data = TEENSCAN)

TEENSCAN <- TEENSCAN %>%
  mutate(SSBTotal_change = SSBTotalPost - SSBTotalPre)

TEENSCAN %>%
  group_by(Group) %>%
  summarise(
    SSBmean_change = mean(SSBTotal_change, na.rm = TRUE),
    SSBsd_change   = sd(SSBTotal_change, na.rm = TRUE),
    n           = sum(!is.na(SSBTotal_change))
  )

t.test(SSBTotal_change ~ Group, data = TEENSCAN)





# ANCOVA ------------------------------------------------------------------------------------------
summary(lm(NKPost ~ Group + NKPre, data = TEENSCAN))
summary(lm(MotPost ~ Group + MotPre, data = TEENSCAN))
summary(lm(FVTotalPost ~ Group + FVTotalPre, data = TEENSCAN))
summary(lm(FruitTotalPost ~ Group + FruitTotalPre, data = TEENSCAN))
summary(lm(VegTotalPost ~ Group + VegTotalPre, data = TEENSCAN))
summary(lm(SSBTotalPost ~ Group + SSBTotalPre, data = TEENSCAN))


# Nutrition Knowledge
m_nk <- lm(NKPost ~ Group + NKPre, data = TEENSCAN)
confint(m_nk, "Group")

# Motivation
m_mot <- lm(MotPost ~ Group + MotPre, data = TEENSCAN)
confint(m_mot, "Group")

# Total Fruit & Veg
m_fv <- lm(FVTotalPost ~ Group + FVTotalPre, data = TEENSCAN)
confint(m_fv, "Group")

# Fruit
m_fruit <- lm(FruitTotalPost ~ Group + FruitTotalPre, data = TEENSCAN)
confint(m_fruit, "Group")

# Vegetables
m_veg <- lm(VegTotalPost ~ Group + VegTotalPre, data = TEENSCAN)
confint(m_veg, "Group")
```

```
# SSB
m_ssb <- lm(SSBTotalPost ~ Group + SSBTotalPre, data = TEENSCAN)
confint(m_ssb, "Group")



# Composite diet ------------------------------------------------------------------------------------------

TEENSCAN <- TEENSCAN %>%
  mutate(
    DietPre  = FruitTotalPre + VegTotalPre - SSBTotalPre,
    DietPost = FruitTotalPost + VegTotalPost - SSBTotalPost,
    DietChange = DietPost - DietPre,
    NKChange = NKPost - NKPre
  )
m_diet <- lm(DietChange ~ NKChange + DietPre, data = TEENSCAN)
summary(m_diet)



ANCOVA
m_diet <- lm(DietPost ~ Group + DietPre, data = TEENSCAN)
summary(m_diet)




# Demographics - Fishers ----------------------------------------------------------------------------------


library(readxl)
CalFresh_Demos <- read_excel("~/Desktop/CalFresh Demos.xlsx")
View(CalFresh_Demos)

fisher.test(table(CalFresh_Demos$`Race/Ethnicity`, CalFresh_Demos$Group))
fisher.test(table(CalFresh_Demos$`Gender Identity`, CalFresh_Demos$Group))
fisher.test(table(CalFresh_Demos$`Age`, CalFresh_Demos$Group))
fisher.test(table(CalFresh_Demos$`BMI`, CalFresh_Demos$Group))
fisher.test(table(CalFresh_Demos$`Tobacco Use`, CalFresh_Demos$Group))
```
